# *In-Situ* High-Resolution Transmission Electron Microscopy Investigation of Overheating of Cu Nanoparticles

**DOI:** 10.1038/srep19545

**Published:** 2016-01-20

**Authors:** Chunlin Chen, Ziyu Hu, Yanfen Li, Limin Liu, Hirotaro Mori, Zhangchang Wang

**Affiliations:** 1 Advanced Institute for Materials Research, Tohoku University, 2-1-1 Katahira, Aoba-ku, Sendai 980-8577, Japan; 2 Beijing Computational Science Research Center, No. 3 He-Qing Road, Hai-Dian District, Beijing 100084, China; 3 Institute for Materials Research, Tohoku University, 2-1-1 Katahira, Aoba-ku, Sendai 980-8577, Japan; 4 Research Center for Ultra-High Voltage Electron Microscopy, Osaka University, 7-1 Mihogaoka, Ibaraki, Osaka 567-0047, Japan

## Abstract

Synthesizing and functionalizing metal nanoparticles supported on substrates is currently the subject of intensive study owing to their outstanding catalytic performances for heterogeneous catalysis. Revealing the fundamental effect of the substrates on metal nanoparticles represents a key step in clarifying mechanisms of stability and catalytic properties of these heterogeneous systems. However, direct identification of these effects still poses a significant challenge due to the complicacy of interactions between substrates and nanoparticles and also for the technical difficulty, restraining our understanding of these heterogeneous systems. Here, we combine *in situ* high-resolution transmission electron microscopy with molecular dynamics simulations to investigate Cu nanoparticles supported on graphite and Cu_2_O substrates, and demonstrate that melting behavior and thermal stability of Cu nanoparticles can be markedly influenced by substrates. The graphite-supported Cu nanoparticles do not melt during annealing at 1073 K until they vanish completely, i.e. only the sublimation occurs, while the Cu_2_O-supported Cu nanoparticles suffer melting during annealing at 973 K. Such selective superheating of the Cu nanoparticles can be attributed to the adsorption of a thin carbon layer on the surface of the Cu nanoparticles, which helps guide further stability enhancement of functional nanoparticles for realistic applications.

Substrate-supported metal nanoparticles represent one of the main heterogeneous catalysts and are being hotly investigated for applications in a wide range of chemical reactions^1^,^2^,^3^, since nanometerials often exhibit unusual properties^4^,^5^. To optimize their catalytic performances, much effort has been devoted to seeking the critical factors influencing catalytic properties of the nanoparticles, such as their particle size, morphology, and the substrates of choice^6^,^7^,^8^,^9^,^10^. It is currently well documented that catalytic properties of the nanoparticles could be largely enhanced by downsizing the nanoparticles so as to increase the surface-to-volume ratio and also by manipulating a specific exposure plane of the nanoparticles^11^,^12^,^13^. In all these circumstances, the interactions between the metal nanoparticles and their substrates, which can affect not only the nanoparticles but also catalytic functionality of the whole system, assume paramount importance. However, our knowledge of such interactions still has not well developed yet due to the complicacy in directly probing them, thereby restraining our fundamental understanding of how substrates can impose an impact on physical and chemical properties of metal nanoparticles.

One of the commonly occurring issues in realistic application of nanoparticles lies in melting of metal nanoparticles. This phenomenon shall be especially relevant in cases where stability of the functional nanoparticles is affected, necessitating the fundamental investigation of melting behavior and thermal stability of substrate-supported nanoparticles^7^,^11^,^14^,^15^,^16^. To this end, we combined *in situ* high-resolution transmission electron microscopy (HRTEM) observations with molecular dynamics (MD) simulations, aimed to conduct a comprehensive study on substrate effect on melting behavior and thermal stability of Cu nanoparticles supported on graphite and Cu_2_O substrates. We provide evidence to the fundamental influence of substrates on melting behavior and thermal stability of Cu nanoparticles, and demonstrate that the graphite-supported Cu nanoparticles can be overheated by retaining crystalline at a temperature that is 100 K higher than the melting point of Cu_2_O-supported Cu nanoparticles.

Figure 1 shows a series of HRTEM images, which reveal a typical annealing process of a Cu nanoparticle on graphite substrate at 1073 K. (see a movie given in Supplementary Movie 1). Note that due to the fast imaging technique (~0.1 s per frame) used when recording the movie, the images are somewhat noisy. At the initial stage (Fig. 1a), one can confirm that the spherical Cu nanoparticle of 2.1 nm (i.e. half of the height) sits on the graphite substrate. The fringes with a spacing of 0.208 nm correspond to {111} lattices of Cu, and those with a spacing of 0.335 nm in the substrate correspond to {0002} lattices of graphite, verifying a successful deposition of Cu nanoparticles on graphite. The Cu nanoparticle gradually shrinks by subsequent annealing, yet retains crystalline, i.e. the sublimation occurs (Fig. 1b,c), which eventually results in the disappearance of the nanoparticle (Fig. 1d). Interestingly, the lattice fringes of Cu nanoparticle can be clearly resolved in the entire shrinking process, implying that the nanoparticle is always in its crystalline form until disappeared completely, i.e. no melting takes place. A similar annealing process is also found for a Cu nanoparticle with radius of ~4 nm on graphite at 1073 K (Supplementary Fig. S1).

To further examine the substrate effect, we prepared Cu nanoparticles by performing an *in situ* electron irradiation of Cu oxides supported on a carbon grid^17^,^18^,^19^,^20^,^21^. As reported in these literatures, the precipitation of metals from their oxides is due to the forced atom displacement which includes the knock-on collision of incident electrons and the radiolytic damage process. The heating effect of electron beam is not important for the decomposition of the metal oxides. Figure 2a shows a representative HRTEM image of a Cu nanoparticle precipitated from Cu oxides by electron irradiation at 973 K. (A movie showing the precipitation of Cu nanoparticles from Cu oxides by electron irradiation is given in Supplementary Movie 2). The fringes with a spacing of 0.301 nm correspond to {110} lattices of Cu_2_O, indicating that the substrate is crystalline Cu_2_O. However, the precipitated Cu nanoparticle shows a perfect semi-spherical morphology with a radius of 4.3 nm, and its image contrast is uniform and absent of any lattice fringes, indicating that the Cu nanoparticle is in liquid state. Figure 2b shows a halo ring taken from the Cu nanoparticle, confirming the liquid state of Cu nanoparticle. The melting point of the Cu nanoparticle on Cu_2_O is hence determined to be no higher than 973 K. Such a melting of Cu nanoparticle on Cu_2_O substrate at 973 K can be confirmed in Supplementary Fig. S2. To extract chemical information, we also conducted an x-ray energy dispersive spectroscopy (EDS) analysis. Figure 2c shows EDS spectrum of the Cu nanoparticle, from which the FCC-Cu peaks are detected solely, implying that the nanoparticle is composed of pure Cu. However, both the Cu and O peaks can be identified in the EDS spectrum of the substrate (Fig. 2d), confirming that the substrate comprise Cu oxide. A HRTEM image showing the crystalline Cu nanoparticle after cooling down to room temperature is given in Supplementary Fig. S3, which clearly presents the lattice fringes of *fcc* Cu.

It is necessary to discuss the heating effect of the electron beam in the experiments shown in Figs 1 and 2. Since the supporting grids in these two experiments are both composed of carbon, the temperature rise due to the radiation of electron beam mainly depends on the thermal conductivity of carbon and the dose current of electron beam. According to the calculation by R. F. Egerton *et al.*^22^, the temperature rise of carbon film under the radiation of 200 kV electron beam is less than 2 K. Obviously, this very small temperature rise can be neglected in the present two experiments.

To date, several melting models can be used to address melting behavior of a nanoparticle^23^,^24^. Among these models, the homogeneous melting model (HMM) which assumes that a particle melts completely with no premelting at the melting temperature, could allow us to extract information on the highest melting temperature for a particle. In terms of the HMM theory, the melting temperature (*T*_m_) for a small particle can be expressed as a function of particle size^23^:


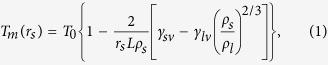


where *T*_0_ is bulk melting temperature (Cu: 1356 K), *r*_s_ is radius of a solid particle, *L* is latent heat (Cu: 2.05 × 10^5^ Jkg^−1^)^25^, *γ*_sv_ and *γ*_lv_ are specific surface energies of solid (Cu: 1.78 Jm^−2^) and liquid (Cu: 1.3 Jm^−2^), respectively^26^, and *ρ*_s_ and *ρ*_l_ are densities of solid (Cu: 8.24 × 10^3^ kgm^−3^) and liquid (Cu: 7.88 × 10^3^ kgm^−3^) at melting point, respectively^27^. From this equation, the *T*_m_ can be calculated with respect to the reciprocal of radius of the particle (see blue line in Fig. 3). Evidently, the melting temperature of a Cu nanoparticle on the Cu_2_O (≤973 K) is found to be lower than that predicted with the HMM. Interestingly, the calculated melting temperature is always lower than the annealing temperature of the Cu nanoparticle on graphite (see red line in Fig. 3). This offers further evidence that the Cu nanoparticle supported on graphite is overheated by tens of K above the melting point predicted by the HMM theory.

To gain insights into structural and physical mechanism of the extremely high thermal stability of the graphite-supported Cu nanoparticle, we first performed aberration-corrected scanning TEM (STEM) analyses to resolve spatially and identify chemically the Cu nanoparticle. Fig. 4a,b presents high-angle annular-dark-field (HAADF) and simultaneously collected annular-bright-field (ABF) STEM images of a typical graphite-supported Cu nanoparticle. Clearly, the Cu nanoparticle shows no sign of oxidation and is covered by a thin amorphous layer. To identify chemically the amorphous skin, we further conducted electron energy-loss spectroscopy (EELS) mapping of the Cu-L and C-K edge, as shown in Fig. 4d,e. By comparing the HAADF image (Fig. 4c) with EELS mapping (Fig. 4d,e), we find that the amorphous skin of the graphite-supported Cu nanoparticle can be identified as carbon with a thickness is ~1 nm, indicating that the covered C may play a pivotal role in stabilizing the Cu nanoparticle.

To test this scenario, we carried out molecular dynamics simulations with two sets of models that can simulate the experimental condition well. One model represents surface clean spherical Cu nanoparticle with a radius of 1.5 nm (Fig. 5a,b) and the other describes the Cu nanoparticle covered with 0.1 nm-thick amorphous C (Fig. 5e,f). The solution of C in Cu is not considered here because the maximum solubility of C in Cu at 1073 K is extremely low (i.e. ~0.01 at. %), as can be seen in the Cu-C phase diagram shown in Supplementary Fig. S4. To simulate melting process, the models undergo a gradual heat treatment from 300 to 1090 K, as shown in Fig. 5. One can notice that the pure Cu nanoparticle starts to melt at its surface (Fig. 5c) and extends to its interiors with the rise of temperature. It eventually melts fully at 1012 K (Fig. 5d). Interestingly, the Cu nanoparticle skinned with C remains crystalline even at 1012 K, indicating a higher melting temperature than the pure Cu nanoparticle (Fig. 5g). However, Cu atoms in the Cu nanoparticle with C are randomly distributed with no lattice fringes at 1052 K, indicating a complete melting (Fig. 5h). The calculated radial pair distribution functions g(r) of Cu nanospheres with/without C skin are shown in Supplementary Fig. S5. As seen in Fig. S5a, the pure Cu nanosphere (i.e. with no C skin) still has three characteristic peaks of Cu at 1000 K and shows characteristics of a liquid phase at 1012 K and 1052 K. On the other hand, the Cu nanosphere with C skin retains the three characteristic peaks of solid Cu at 1000 K and 1012 K and shows characteristics of a liquid phase at 1052 K, suggesting that the melting point of the Cu nanosphere with C skin is 1052 K.

To determine exact melting point for the Cu nanoparticle, we calculated potential energy of the two systems, as shown in in Fig. 6. An abrupt change in potential energy is visible at 1012 K for the pristine Cu nanoparticle, indicating that it has a melting point of 1012 K, consistent well with the previous reports^28^,^29^. On the other hand, there appears a similar abrupt change in potential energy at 1052 K for the Cu nanoparticle with C, suggesting that its melting point is 40 K higher than that of the pristine nanoparticle, in support of the observed overheating of the Cu nanoparticle with C. It is noted that although the theoretical melting points deviate from their experimental counterparts, the trend for the two types of nanoparticles is consistent, offering a qualitative support.

Probing melting behavior and thermal stability of metal nanoparticles can not only deepen our understanding of the melting nature of a particle, but facilitate their application at realistic technical conditions. Our results provide clear evidence that substrate can play a fundamental role in affecting the melting behavior and thermal stability of the supported Cu nanoparticles. Particularly, we have demonstrated that the graphite-supported Cu nanoparticle can be overheated by retaining crystalline at a temperature of 100 K higher than the melting point of the Cu_2_O-supported Cu nanoparticles. Further molecular dynamics simulations attribute the significantly enhanced thermal stability of the Cu nanoparticles to the adsorption of a thin C layer at their surface. Although demonstrated with Cu nanoparticles, the findings on the substrate effect and the overheating of the Cu nanoparticles could in principle be applied to understand other substrate-supported metal nanoparticles, opening thereby a novel avenue in enhancing thermal stability of substrate-supported metal nanoparticles in general for practical technological applications.

## Methods

### TEM sample preparation and microscopic characterization

The Cu nanoparticles supported on the graphite substrates were fabricated in Hitachi H-800 type 200-kV transmission electron microscope equipped with an evaporator. The graphite film characterized by a flake of highly oriented pyrolytic graphite was mounted into the Gatan single-tilt (Model 628) heating holder and baked at 1273 K for 10 min in the microscope in order to obtain a clean surface. Once the graphite film was cooled to room temperature, high-purity copper (99.99 wt.%) was evaporated onto the graphite substrate in the microscope with a base pressure of 10^−5^ Pa, forming the Cu nanoparticles. The Cu nanoparticles and the heating holder were then transferred instantly to the Hitachi HF-2000 type 200-kV HRTEM. The annealing was carried out under a high vacuum with a pressure of lower than 10^−6^ Pa. The Cu nanoparticles on the graphite film were slowly heated up to 1073 K at a heating rate of ~1 Ks^−1^ and then maintained at this temperature. The phase transition and morphological transformation of Cu nanoparticles during annealing were monitored *in situ* using supersensitive charge-coupled device camera (AMT, XR-60BFE) at a frame rate of 9 fps. The HAADF, ABF images, and EELS mapping were obtained using the 200-kV STEM (JEM-ARM200F, JEOL) equipped with a probe corrector (CEOS, Gmbh), which offers an unprecedented opportunity to probe structures with sub-ångström resolution.

### Calculation methodology

Molecular dynamics (MD) simulation was conducted by the Large-scale Atomic/Molecular Massively Parallel Simulator (LAMMPS) package^30^. For the pure Cu system, the radius of the Cu spherical particle was adopted to be 1.5 nm, and the interlayer Cu-Cu spacing was adopted to be 0.221 nm. On the other hand, for the C/Cu systems, the Cu spherical particle with a radius of 1.5 nm was wrapped by an amorphous carbon layer with thickness of 0.1 nm. The spacing between the outmost Cu layer and the surrounded C layer was adopted as 0.3 nm. The Cu-Cu and C-C interactions were described by Embedded-Atom Method (EAM)^31^ and Adaptive Intermolecular Reactive Empirical Bond Order (AIREBO)^32^, respectively. The Cu-C interactions were described by the LJ (12-6) potential. The simulation was initiated by placing the Cu sphere and C/Cu sphere in a box with a dimension of 8 nm × 8 nm × 8 nm. MD simulations were performed with the canonical (NVT) ensemble. The Nosé-Hoover thermostat was tuned by adjusting temperature of the thermal bath *via* adding an extra degree of freedom to the Hamiltonian^33^. The systems were first relaxed at 300 K using the NVT ensemble for a sufficient period of time (30 ps). The sample was then allowed to evolve for 15 ps at the temperature ranging from 900 K to 1090 K. Data were collected once the simulation reached an equilibrium state (10 ps after the initiation of the simulation).

## Additional Information

**How to cite this article**: Chen, C. *et al.* In-Situ High-Resolution Transmission Electron Microscopy Investigation of Overheating of Cu Nanoparticles. *Sci. Rep.* **6**, 19545; doi: 10.1038/srep19545 (2016).

## Supplementary Material

Supplementary movie 1

Supplementary movie 1

Supplementary Information

## Figures and Tables

**Figure 1:**
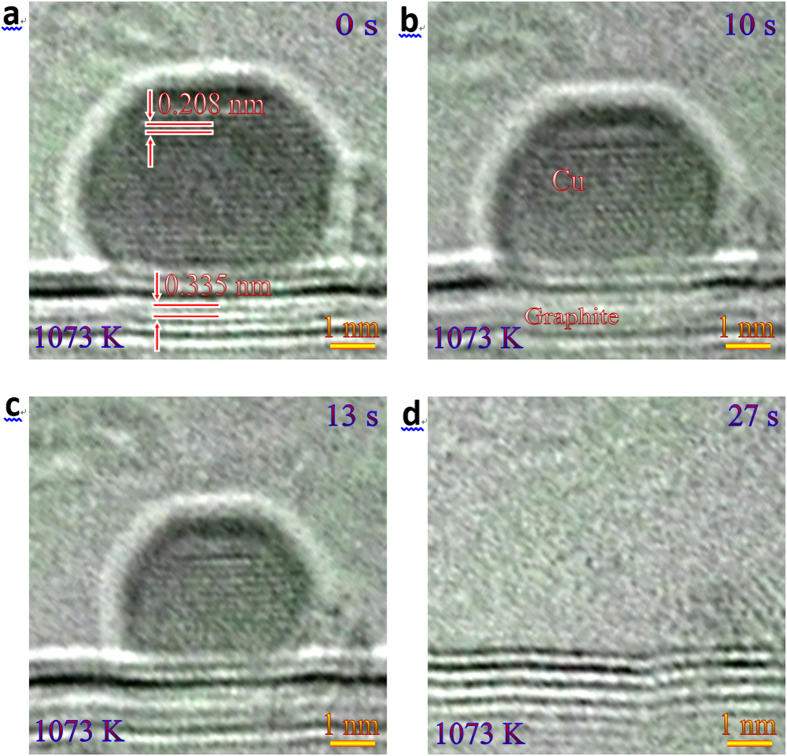
Annealing process of a Cu nanoparticle on the graphite. A series of HRTEM images as a function of elapsed time showing the whole annealing process of a Cu nanoparticle on graphite at 1073 K: (**a**) 0 s, (**b**) 10 s, (**c**) 13 s, and (**d**) 27 s. The lattice fringes of Cu nanoparticle can be clearly identified in the entire shrinking process, suggesting that the nanoparticle keeps crystalline without any melting until it disappears completely.

**Figure 2:**
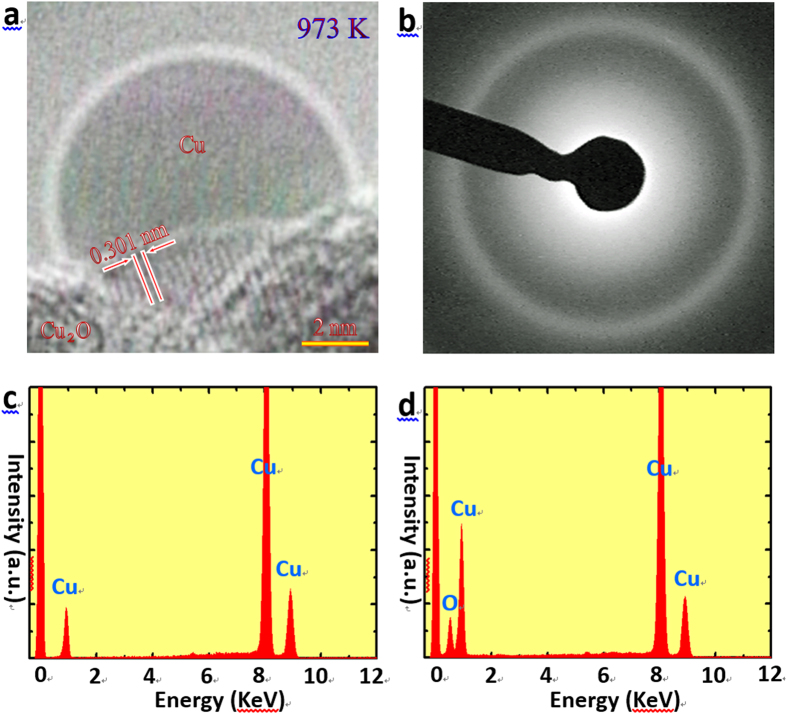
A Cu nanoparticle on the Cu oxide. (**a**) A HRTEM image taken at 973 K revealing a liquid Cu particle precipitated from Cu oxides by the *in situ* electron irradiation at 973 K. (**b**) An electron diffraction pattern showing the halo ring of liquid Cu. (**c,d**) EDS spectra of the nanoparticle (**c**) and the substrate (**d**).

**Figure 3:**
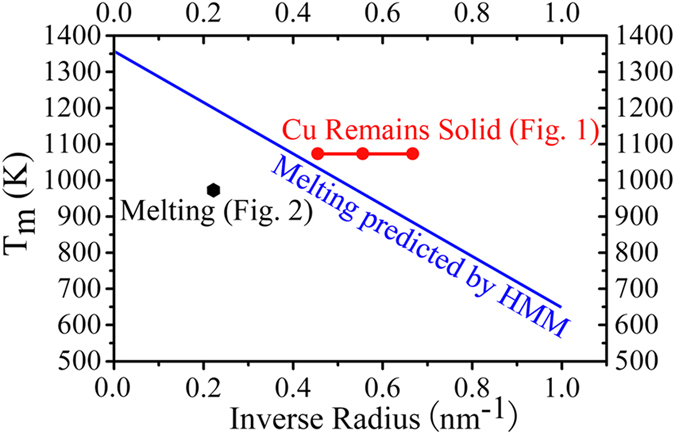
Melting temperature as a function of the radius of the Cu nanoparticles. The melting temperature of Cu nanoparticles predicted by the homogeneous melting model (HMM) is given by the blue line.

**Figure 4:**
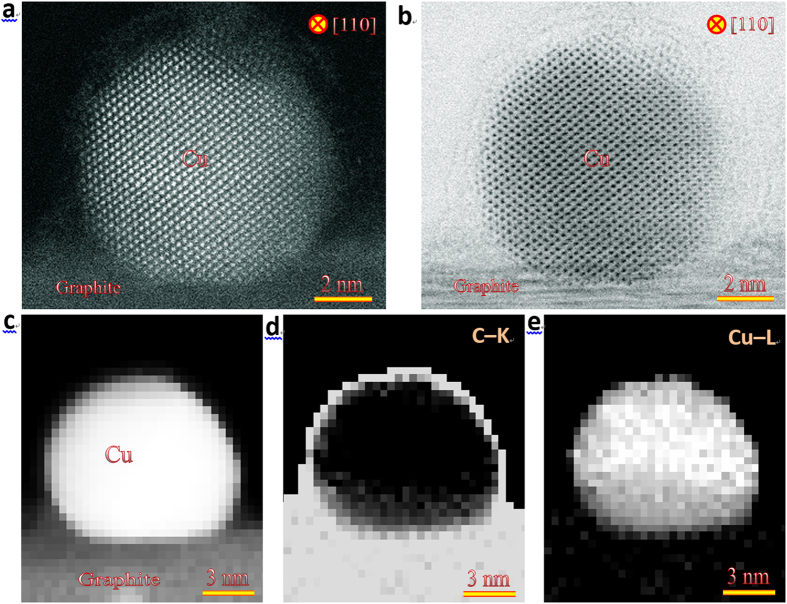
Imaging and chemical identification of the graphite-supported Cu nanoparticles. (**a**) HAADF and (**b**) ABF images of a typical Cu nanoparticle supported on the graphite. There appears a thin amorphous layer on the surface of otherwise pure Cu nanoparticle. (**c**)–(**e**) HAADF STEM (**c**) and core-loss images of C-K (**d**) and Cu-L (**e**) edges for a graphite-supported Cu nanoparticle. The amorphous layer with a thickness of ~1 nm on the surface of Cu nanoparticle is identified as C.

**Figure 5:**
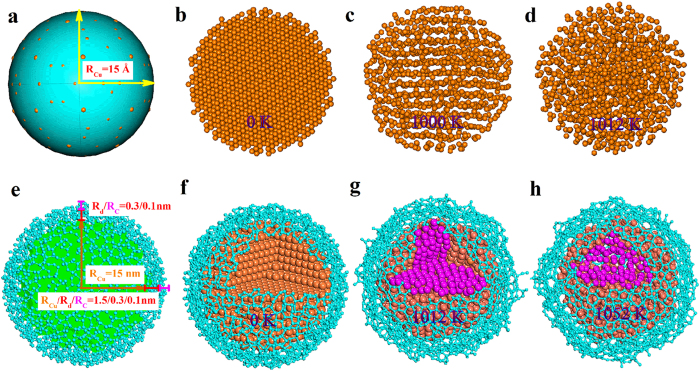
Structural models revealing the melting progress of Cu nanospheres. (**a**,**e**) Schematic diagrams showing the models without (**a**) and with (**e**) C skin used for the MD simulations. The *R*_Cu_, *R*_d_, and *R*_C_ denote the radius of the simulated Cu sphere, the distance between Cu sphere and C, and the thickness of C, respectively. (**b–d**) Structural models for the Cu nanoparticle without the C skin obtained at 0 K (**b**), 1000 K (**c**), and 1012 K (**d**). (**f–h**) Structural models for the Cu nanoparticle covered with C obtained at 0 K (**f**), 1012 K (**g**), and 1052 K (**h**).

**Figure 6:**
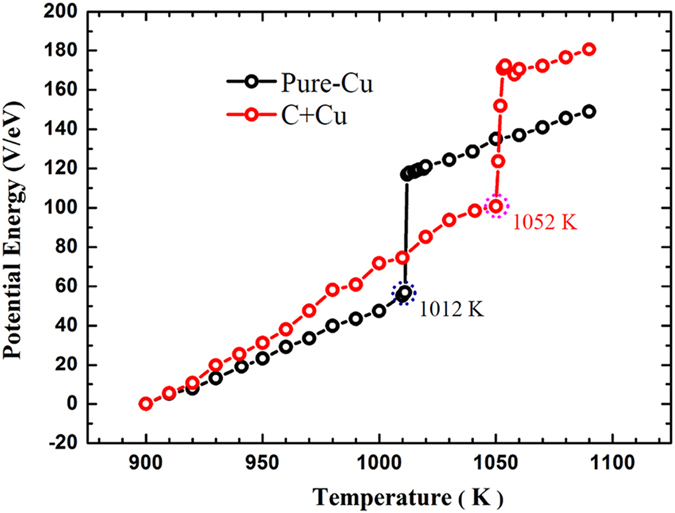
Potential energy as a function of temperature. The black circles indicate the potential energy for the pure Cu nanoparticle, and the red ones that of the Cu nanoparticle covered by C. The melting points in each case are marked by dashed circles: 1012 K for the pure Cu nanoparticle, and 1052 K for the Cu nanoparticle covered by C. The Cu nanoparticle with C exhibits a higher melting point than the pure Cu nanoparticle, implying that the covered C plays a relevant role in affecting the melting temperature of the nanoparticle.
