# Supplementary material for: In-Situ High-Resolution Transmission Electron Microscopy Investigation of Overheating of Cu Nanoparticles
Source: Sci Rep. 2016 Jan 20;6:19545. doi: 10.1038/srep19545 (PMC4726356; doi:10.1038/srep19545)
Supplement: Supplementary Information [file srep19545-s3.doc]

Supplementary Information for

**In-Situ High-resolution Transmission Electron Microscopy Investigation of Overheating of Cu Nanoparticles**

Chunlin Chen^1†^, Ziyu Hu^2†^, Yanfen Li^3†^, Limin Liu^2^, Hirotaro Mori^4^ & Zhangchang Wang^1^

^1^*Advanced Institute for Materials Research, Tohoku University, 2-1-1 Katahira, Aoba-ku, Sendai 980-8577, Japan*

^2^*Beijing Computational Science Research Center, No. 3 He-Qing Road, Hai-Dian District, Beijing 100084, China*

^3^*Institute for Materials Research, Tohoku University, 2-1-1 Katahira, Aoba-ku, Sendai 980-8577, Japan*

^4^*Research Center for Ultra-High Voltage Electron Microscopy, Osaka University, 7-1 Mihogaoka, Ibaraki, Osaka 567-0047, Japan*

**Supplementary Figure S1**

**b**


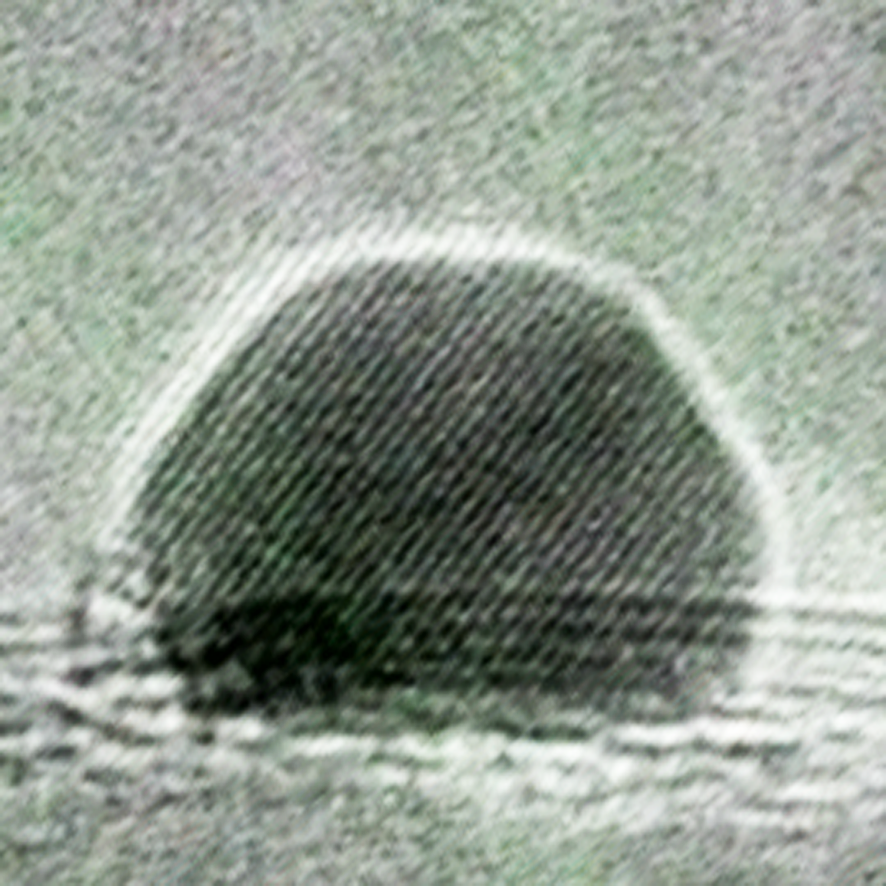


**c**


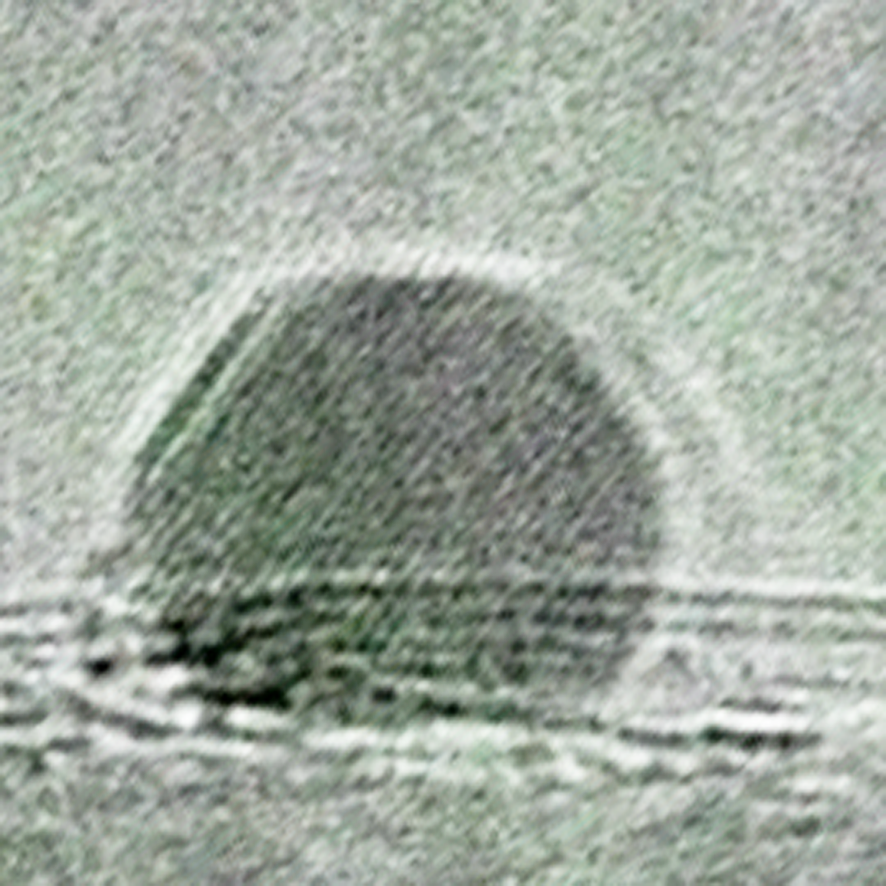


**d**


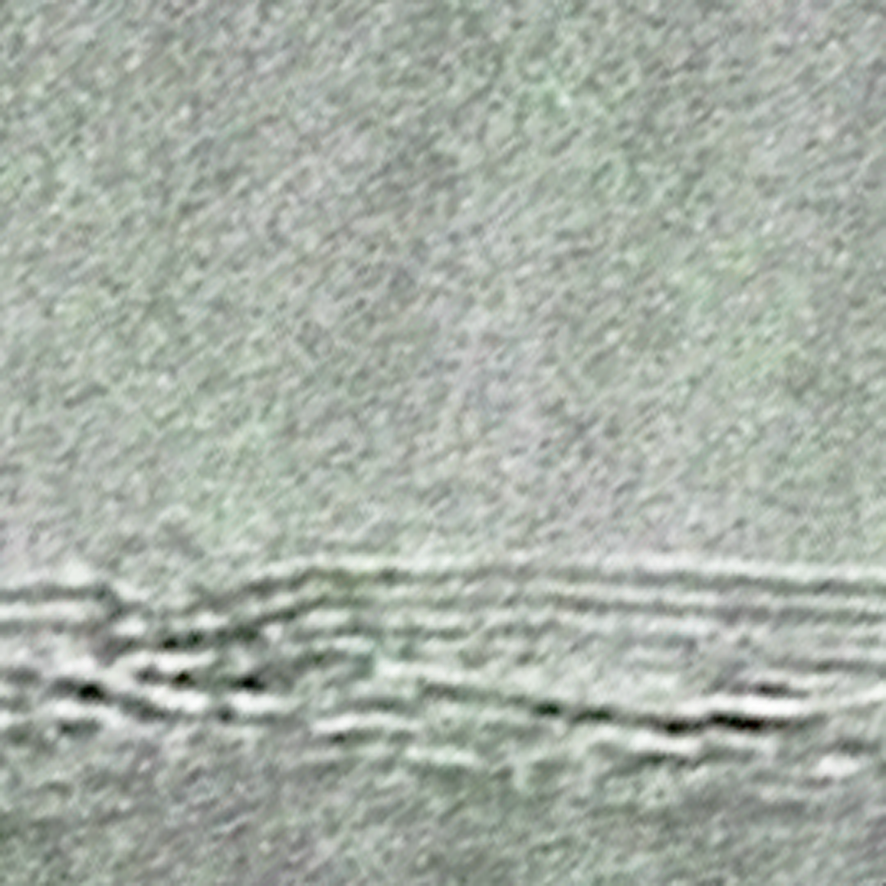


**a**


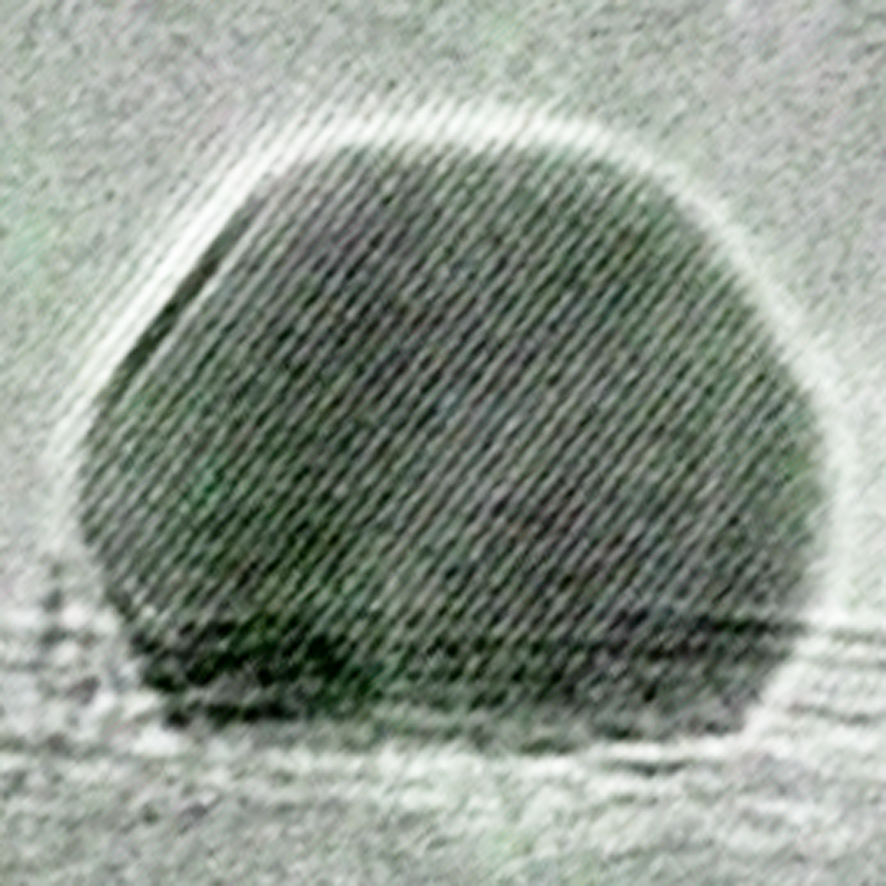


**Figure S1|Annealing process of a Cu nanoparticle on the graphite.** HRTEM images as a function of elapsed time showing the whole annealing process of a Cu nanoparticle on graphite at 1073 K: (**a**) 0 s, (**b**) 4 s, (**c**) 5 s, and (**d**) 6 s. Lattice fringes of Cu nanoparticle can be clearly identified during the entire shrinking process, implying that the nanoparticle keeps crystalline without any melting until it disappears completely.

**Supplementary Figure S2**

**c**


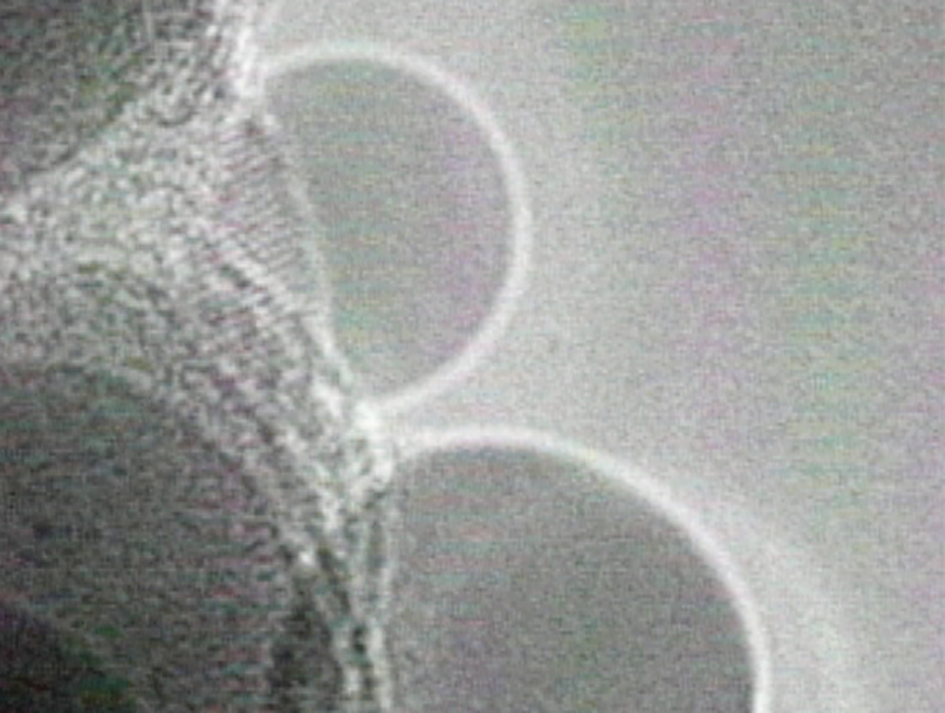


**d**


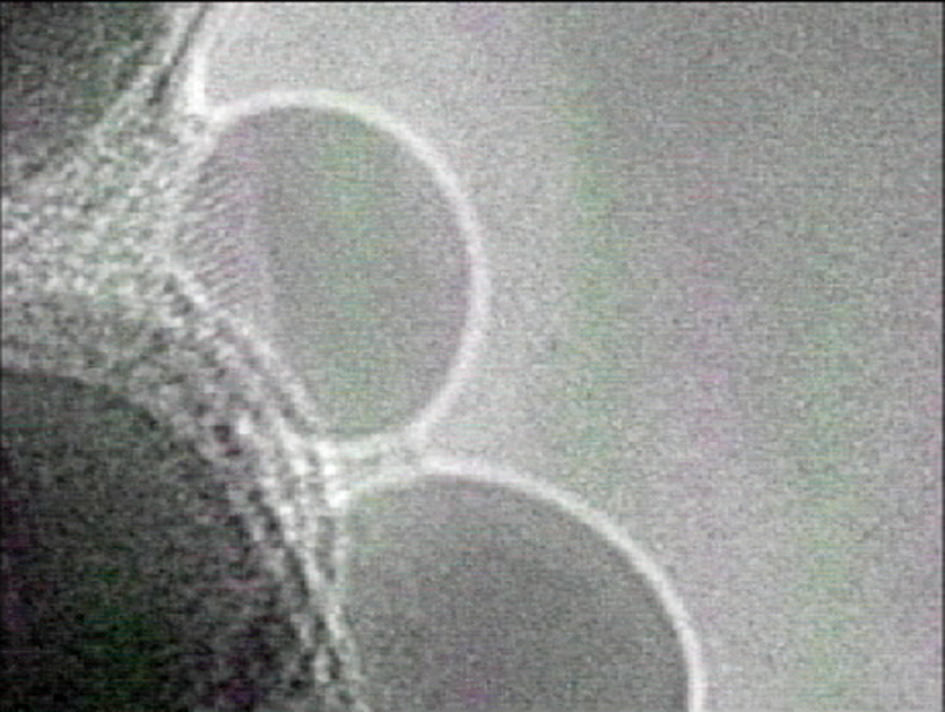


**a**


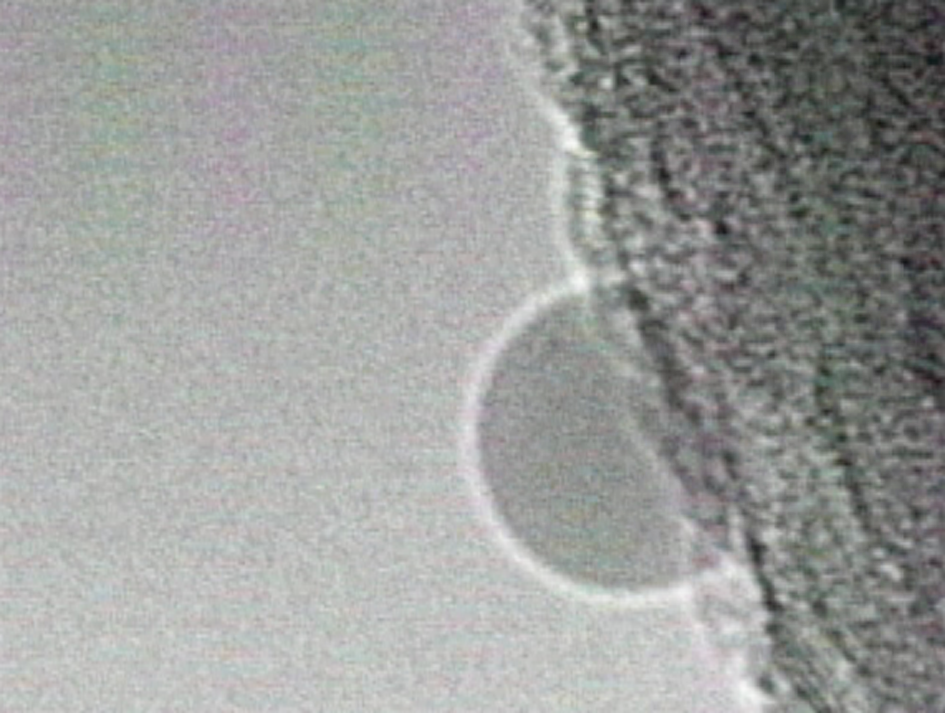


**b**


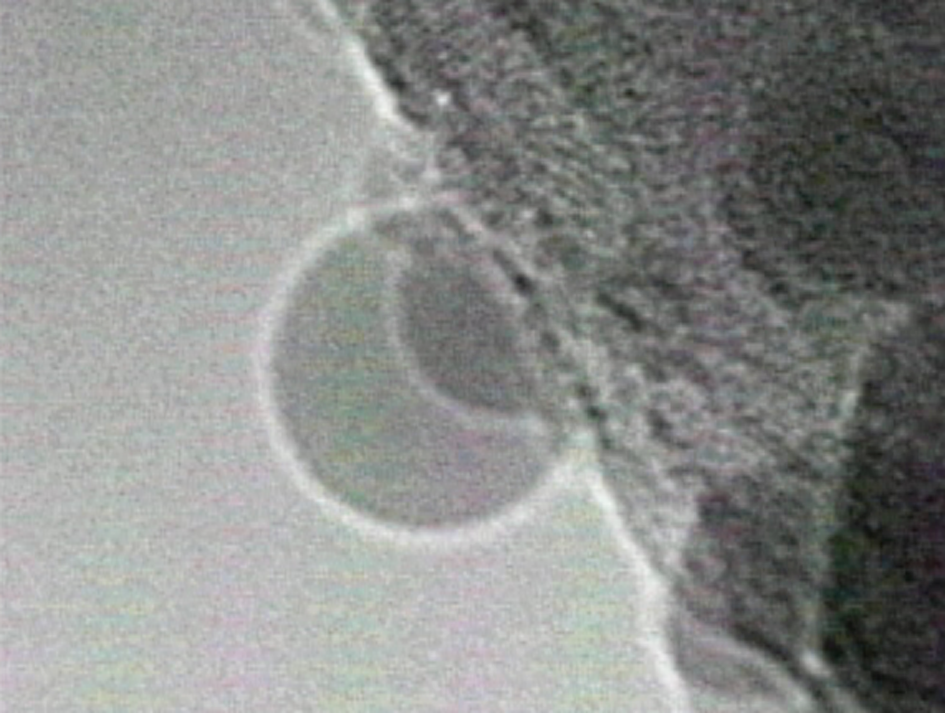


**Figure S2|Cu nanoparticles on the Cu oxide.** HRTEM images taken at 973 K revealing the liquid Cu particles precipitated from Cu oxides by *in situ* electron irradiation at 973 K.

**Supplementary Figure S3**


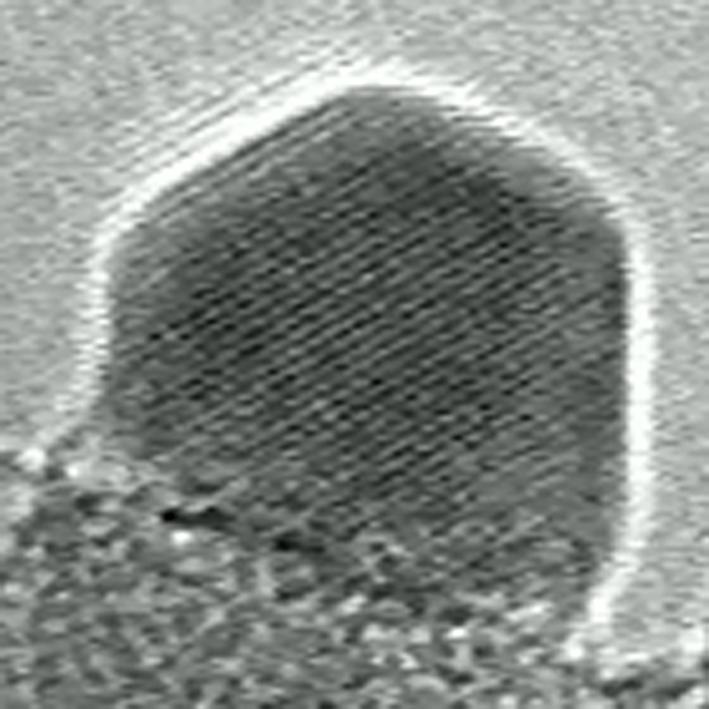


**Figure S3|HREM image showing the crystalline Cu nanoparticle after cooling down to room temperature**.

**Supplementary Figure S4**


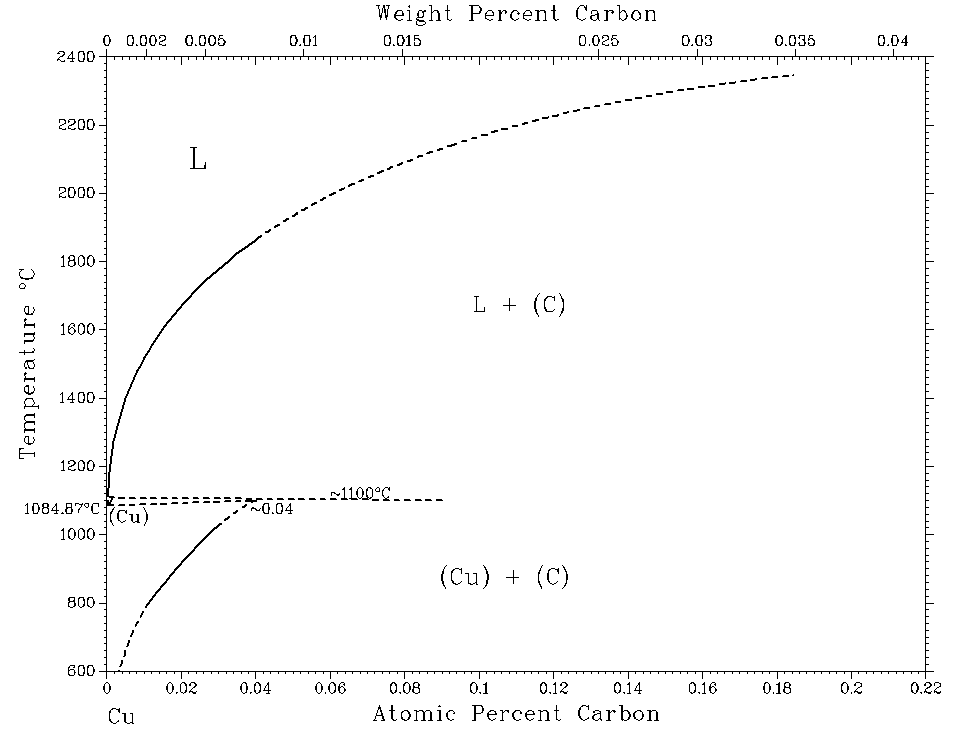


**Figure S4|Cu-C phase diagram**. The maximum solubility of C in Cu at 1073 K is extremely low (i.e. ~ 0.01 at. %)^1^.

**Supplementary Figure S5**

**Figure S5|Calculated radial pair distribution functions g(r).** (a) Cu nanosphere without C skin, (b) Cu nanosphere with C skin.

**Supplementary References**

1. Massalski, T. B., Okamoto, H. Subramanian, P. R. & Kacprzak, L. Binary Alloy Phase Diagrams. Ohio, USA: ASM International; 1990.
